# 3D Culture Modeling of Metastatic Breast Cancer Cells in Additive Manufactured Scaffolds

**DOI:** 10.1021/acsami.2c07492

**Published:** 2022-06-10

**Authors:** Afroditi Nanou, Ivan Lorenzo-Moldero, Kyriakos D. Gazouleas, Barbara Cortese, Lorenzo Moroni

**Affiliations:** † Tissue Regeneration Department, MIRA Institute for Biomedical Technology, University of Twente, Drienerlolaan 5, 7522 ND Enschede, The Netherlands; ‡ Medical Cell BioPhysics Department, Faculty of Science and Technology, University of Twente, Dienstweg 1, 7522 ND Enschede, The Netherlands; § Complex Tissue Regeneration Department, MERLN Institute for Technology-Inspired Regenerative Medicine, Maastricht University, Universiteitssingel 40, 6229 ER Maastricht, The Netherlands; ∥ National Research Council-Nanotechnology Institute (CNR Nanotec), 00185 Rome, Italy

**Keywords:** scaffolds, tissue engineering, tumor microenvironment, three-dimensional bioprinting, breast cancer

## Abstract

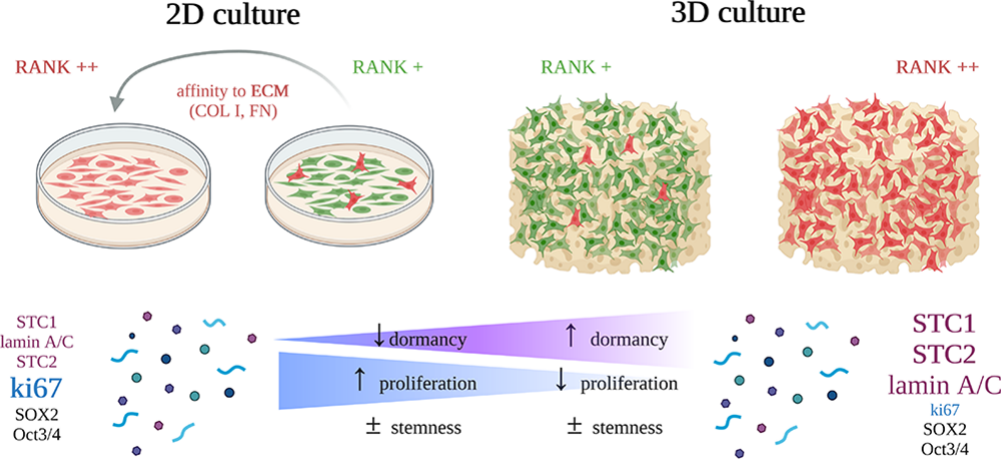

Cancer biology research is increasingly moving toward innovative *in vitro* 3D culture models, as conventional and current 2D cell cultures fail to resemble *in vivo* cancer biology. In the current study, porous 3D scaffolds, designed with two different porosities along with 2D tissue culture polystyrene (TCP) plates were used with a model breast cancer human cell line. The 3D engineered system was evaluated for the optimal seeding method (dynamic versus static), adhesion, and proliferation rate of MDA-MB-231 breast cancer cells. The expression profiles of proliferation-, stemness-, and dormancy-associated cancer markers, namely, ki67, lamin A/C, SOX2, Oct3/4, stanniocalcin 1 (STC1), and stanniocalcin 2 (STC2), were evaluated in the 3D cultured cells and compared to the respective profiles of the cells cultured in the conventional 2D TCP. Our data suggested that static seeding was the optimal seeding method with porosity-dependent efficiency. Moreover, cells cultured in 3D scaffolds displayed a more dormant phenotype in comparison to 2D, which was manifested by the lower proliferation rate, reduced ki67 expression, increased lamin A/C expression, and overexpression of STCs. The possible relationship between the cell affinity to different extracellular matrix (ECM) proteins and the RANK expression levels was also addressed after deriving collagen type I (COL-I) and fibronectin (FN) MDA-MB-231 filial cell lines with enhanced capacity to attach to the respective ECM proteins. The new derivatives exhibited a more mesenchymal like phenotype and higher RANK levels in relation to the parental cells, suggesting a relationship between ECM cell affinity and RANK expression. Therefore, the present 3D cell culture model shows that cancer cells on printed scaffolds can work as better representatives in cancer biology and drug screening related studies.

## Introduction

Worldwide, cancer still represents the second main cause of death. On the basis of the Surveillance Research of the American Cancer Society, it is estimated that, within 2021 only in the US, more than 1.9 × 10^6^ new cases are expected to occur with approximately 0.6 × 10^6^ deaths.^1^ Among the different developing cancer sites in males and females, prostate and breast cancer stand for 26% and 30% of the total cancer cases, respectively, with breast cancer cells preferentially metastasizing to bone, leading to osteolytic lesions, pointing out their urgent need for treatment.^1^ Furthermore, with the current SARS-CoV-2 outbreak, cancer patients experience an increased risk of infection-related fatality due to the underlying malignancy or/and their treatment-related immunocompromised state.^2^

Efforts devoted to the development of effective approaches to cure cancer are conventionally carried out in *in vitro* and *in vivo* model systems; however, these still fail to simulate the pathophysiology of cancer. 2D cancer cell cultures on tissue culture polystyrene (TCP) are easy to perform but they do not represent a physiological culture system, more than often leading to deceiving conclusions.^3^ Even when 2D plates are coated with proteins of the extracellular matrix (ECM), such as laminin, collagen, and fibronectin, the absence of physiological patterns found in living organisms curbs cell adherence and conformation in space, proliferation, signal transduction, migration, and response to therapeutic stimuli.^4^ On the other hand, the use of mice models and xenografts represents a very expensive approach, requiring high costs and expertise for mice manipulation^5^ as well as ethical issue awareness.^6^ In addition, the genotype and biology of stromal cells are different between mice and humans. In recent years, tissue engineering has shown promising results in the fabrication of 3D *in vitro* models able to better capture the complexity of the *in vivo* micro- and macroenvironments.^7^ Various 3D systems have emerged varying in material, structure, procedure, and application.^8^ However, conventional methods for scaffold fabrication, such as electrospinning, freeze-drying, and particle leaching,^9,10^ lack control over the 3D structural design. Biofabrication technologies constitute a strategic platform to develop new *in vitro* culture systems by providing specific control of pore sizes and shapes.^11^ Using PEG-based bioinks,^12^ gelatin–alginate–fibrinogen bioinks,^13^ Matrigel^14^ and alginate,^13^ these techniques have highlighted, over the years, the discrepancies between 2D and 3D microstructures. Despite the advantage of these microenvironments over 2D cultures, few studies have investigated the incorporation of breast cancer associated ECM proteins (such as collagen types I and III, fibronectin, and laminin) to recreate nativelike breast cancer microenvironments.^15^

Proliferation and stemness markers may aid in predicting patient outcome, independently of treatment. Stemness markers such as SOX2 and Oct3/4 are renowned for the regulation of embryogenesis, maintenance of pluripotency, and self-renewal of stem cells.^16^ Furthermore, SOX2 and Oct3/4 have been documented in many different cancer types, suggesting that their high levels are associated with high-grade tumors and a poor prognosis.^17^ STC1 and STC2 are closely related secreted glycoproteins.^18^ While STC1 expression has been correlated with tumor progression as well as metastasis in breast cancer,^19^ STC2 has been shown to be an elusive marker for the prediction of tumor progression.^20^ In fact, its expression has been associated with both poor^21^ and good prognoses in breast cancer patients.^22^ Because of the diverse roles of these biomarkers, it is important to validate their expression within their microenvironment.

In the present study, 3D polyactive (PA) scaffolds were chosen as tissue-engineered culture models. Copolymers such as 300-poly(ethylene oxide)-poly(ethylene oxide) terephthalate 55/polybutylene terephthalate 45 (300PEOT55PBT45 or just PEOT/PBT) (PolyActive) show promising advantages in tissue engineering and drug delivery applications, due to their tunable degradation and higher wettability.^23^ Although they provide a bioinert substrate by reducing effective cell adhesion and viability,^24^ these polymeric biomaterials have reached clinical trials as bone fillers and are under consideration for tissue-engineered clinical treatments of bone and cartilage defects.^9,25,26^ Scaffolds were fabricated via additive manufacturing (AM), allowing a high standardization and tailorable architecture. The choice of the scaffolds allowed the 3D deposition of a synthesized ECM and multiple cell-type cultures.^7,27,28^ The model presented here is based on cells cultured in AM scaffolds to further resemble metastatic bonelike structures. Optimizations of the porosity of the scaffolds and cell seeding were investigated in terms of the adhesion and proliferation rate of MDA-MB-231 breast cancer cells. We compared the localization and protein expression profiles for metastasis-, proliferation-, and dormancy- associated cancer markers between 2D and 3D microenvironments using Western blotting (WB) and ELISA assays. As high expression levels of the receptor activator of NFκB (RANK) of breast cancer cells have been associated with enhanced osteotropism,^29^ we addressed the possible relationship between the ECM cell affinity to different ECM proteins (COL-I or FN) and RANK expression levels. To the best of our knowledge, we reveal for the first time a relation between the cell preference to attach on different ECM proteins and the expression levels of the prognostic marker RANK.

## Materials and Methods

### 3D Scaffold Fabrication and Pretreatment Prior to Cell Seeding

Porous 3D scaffolds were fabricated using a BioScaffolder device (Envisiontec GmbH, Germany), capable of plotting constructs toward each *XYZ* direction, as previously reported.^30^ Briefly, the copolymer poly(ethylene oxide terephthalate)-poly(butylene terephthalate) (PEOT/PBT), referred to hereafter as PA, was placed in a stainless-steel syringe, with needles of about 250 μm internal diameter (ID). The syringe was filled with nitrogen (N_2_) to minimize possible oxidation of the polymer. Thereafter, the polymer was heated above its melting point to a temperature of *T* ≈ 190–195 °C through a heated cartridge unit. After approximately 30 min of heating, a nitrogen pressure of 5 bar (500 kPa) was applied to the syringe through a pressurized cap. Rectangular models were loaded on the BioScaffolder CAD/CAM software and plotted layer by layer through extrusion of the copolymer in a fiber form. The fibers were plotted with a 0–90° pattern, meaning 90° angle steps between two subsequent layers, with a diameter equal to the nozzle diameter d1 = 250 μm. The fiber spacing in the same layer d2 and the layer thickness d3 were set to be d2 = 450 or 650 μm and d3 = 150 μm, respectively.

**Figure 1:**
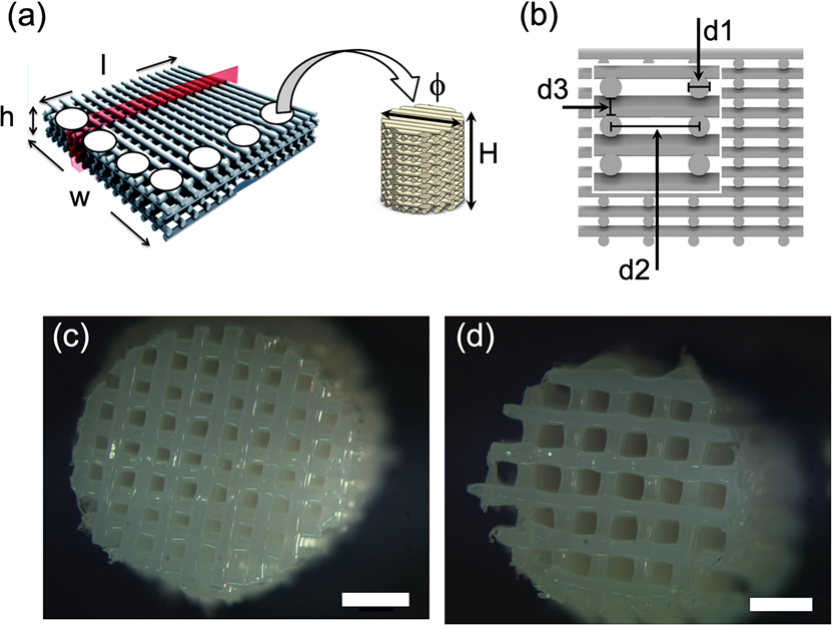
3D scaffold fabrication and characterization. (a) Representative 3D bioplotted PA scaffold block (*w* × *l* × *h* = 20 × 20 × 3 mm, ϕ = 4 mm, *H* = 20 layers). (b) Schematization of a CAD model, illustrating the main parameters defining the scaffold architecture: namely, fiber configuration, fiber diameter d1, fiber spacing d2, and layer thickness d3. The scaffolds used had a 0–90° pattern, d1 = 250 μm, d2= 450 or 650 μm, and d3 = 150 μm. (c, d) Images of two representative scaffold units with the two different porosities used, arising from two different strand distances d2: namely, (c) d2 = 450 μm (small pores/low porosity) and (d) d′2 = 650 μm (big pores/high porosity). Scale bar: 1 mm.

The deposition speed was in the range of 175–225 mm/min to keep a balance between the desired fiber diameter and an overall open porosity. Polymer cylindrical scaffolds of 4 mm diameter were punched out of each block using a biopsy punch, as depicted in Figure 1. Scaffolds were then sterilized in 70% ethanol overnight and washed three times with distilled H_2_O. Finally, scaffolds were coated with an ECM solution (100 μg/mL of COL-I or 20 μg/mL of FN) following immersion in the corresponding coating solution, centrifugation, and incubation at 37 °C overnight.

### MDA-MB-231 Cells

MDA-MB-231 breast cancer cells (American Type Culture Collection, ATCC) were maintained in Dulbecco’s Modified Eagle Medium/Nutrient Mixture F-12 (DMEM/F12) (Gibco, 31331-093, Carlsbad, CA, USA) supplemented with 5% fetal bovine serum (FBS) (Lonza, Verviers, Belgium), 100 U/mL of penicillin and 100 μg/mL of streptomycin (Gibco 15140-122, Carlsbad, CA). Cells were cultured at 37 °C in a humidified atmosphere containing 21% O_2_ and 5% CO_2_, and the culture medium was refreshed every second day.

Subpopulations of MDA-MB-231 were selected by successive “panning” of the initial heterogeneous MDA-MB-231 cell population (parental cells) on 100 μg/mL of COL-I or 20 μg/mL of FN, resulting in the COL-I and FN filial MDA-MB-231 cells. More specifically, tissue culture flasks were coated with 100 μg/mL of type I COL or 20 μg/mL of FN for at least 3 h (or overnight) at 37 °C. MDA-MB-231 cells were plated onto COL-I-or FN-coated flasks at 30000 cells/cm^2^ cell density. After 30 min, nonadherent cells were washed away thoroughly by three successive washing steps with the culture medium. The culture medium was added, and the remaining cells were allowed to expand until they reached 80% confluence. Subsequently, cells were trypsinized and the “panning” procedure was repeated for at least eight passages. The finally derived (filial) cells were used in the experiments.

### Cell Seeding on 3D Scaffolds

Cells were seeded on the scaffolds using either dynamic or static seeding. The two seeding methods were evaluated on the basis of the achieved cell attachment and distribution along the scaffolds after 1 day of culture. Dynamic seeding was carried out by placing each coated scaffold in a 2 mL sterile Eppendorf tube containing the desired cell number suspended in 1.96 mL of culture medium and rocked on a wave motion platform shaker (Heidolph, Polymax 1040) at low speed (15–20 rpm) at 37 °C for 4 h. Static seeding was obtained by placing the scaffolds in nontreated 24-well plates, seeding with 25 μL of a condensed cell suspension containing half of the desired cell number, and incubating at 37 °C for 1 h. After 1 h, each scaffold was turned over. An additional cell suspension of 25 μL was added and the scaffold was incubated for another 1 h. During seeding, 5–7 mL of ddH_2_O was poured between the wells to avoid evaporation of the droplets. At the end, scaffolds were transferred to wells containing 1 mL of the culture medium. The culture medium was refreshed every second day by transferring scaffolds into fresh wells. Cell cultures were placed at 37 °C in a humidified environment with 5% CO_2_ in air and kept for 1, 8, or 10 days depending on the experiment. The density of cells for 3D scaffolds was higher with respect to 2D scaffolds to compensate for the larger surface area, in order to obtain no significant differences in terms of cell densities (around 100000 cells/cm^2^ in all cases) in all culture systems after 8–10 days of cell culturing.

### Methylene Blue Staining

Methylene blue staining was performed to visualize and compare cell attachment and distribution along the scaffolds for the different seeding methods (static and dynamic). Biological duplicates were used for each seeding condition, and different initial cell densities were assessed: namely, 2, 5, and 10 × 10^5^ cells/scaffold for 1 and 7 day(s). Scaffolds were washed once with PBS. Fixation with either 1 mL of 4% paraformaldehyde PFA (Sigma-Aldrich, P6148, St. Louis, MO) or 1 mL of 10% formalin was performed for 30 min. Subsequently, scaffolds were washed three times with ddH_2_O. Scaffolds were then covered with 0.25% w/v methylene blue staining solution (Sigma-Aldrich, M9140, St. Louis, MO) in a 50% v/v ethanol solution for approximately 2 min and thoroughly washed three times with ddH_2_O. Bright-field imaging of the cells was performed using an optical stereomicroscope (Nikon Eclipse TE 300).

### Immunofluorescence (IF) Staining and Imaging

Immunofluorescence (IF) staining was performed at day 8 or 10 in the case of cell cultures on scaffolds in order to have sufficient cells attached on the scaffolds and at day 2 in case of cell cultures on coverslips. Briefly, cells were fixed with either 4% parafolmaldehyde or 10% formalin for 30 min at room temperature. Three washing steps with PBS followed the fixation. At this point, scaffolds were pretreated with filtered 0.1% w/v Sudan Black B solution (Sigma-Aldrich, 199664, St. Louis, MO) in 70% ethanol for 45 min to quench their autofluorescence. Four more washing steps with PBS at room temperature were carried out after Sudan Black pretreatment. Samples were incubated with 0.1% Triton-X and 2% BSA (in PBS) for 15 min at room temperature for cell membrane permeabilization. With RANK staining, which is located on the cell membrane, cells were not permeabilized but only washed with 2% BSA in PBS. Cells were incubated overnight at 4 °C in a respective primary antibody solution. Each primary antibody used was diluted 1:200 in an antibody buffer solution (0.05% v/v Tween-20 and 2% w/v BSA in PBS). After three 5 min washing steps in PBS, cells were incubated for 1 h in the secondary antibody conjugated with Alexa Fluor 488 (Invitrogen, Germany) (1:500) and in Alexa Fluor 568 (Invitrogen, A12380, Germany) (1:100) protected from light. The antibody buffer solution was the same as mentioned above. Two more 5 min washing steps with PBS followed, until cells were incubated for another 10 min at room temperature in DAPI (Sigma-Aldrich, St. Louis, MO) diluted 1:100 in antibody solution. After immunostaining, both scaffolds and coverslips were washed twice with PBS and once with ddH_2_O and mounted in Mowiol (Sigma-Aldrich, St. Louis, MO). Images were acquired using a Nikon Eclipse E600 microscope at wavelengths of 488, 568, and 647 nm, corresponding to the excitation sources of the fluorophores used. Digital images were processed using the software ImageJ v.1.47.

### “Priming” Experiments

“Priming” experiments were included to evaluate the robustness of the model as well as cell plasticity. Cells cultured either in 2D or 3D scaffolds are named “primed” cells, as they are conditioned by the cell culture dimensionality. Microenvironmental factors may, in fact, prime tumor cells toward invasive phenotypes causing a modulation of the biological, biochemical, and/or biophysical factors, in response to reinforcing the aberrant ECM environment.^31,32^ MDA-MB-231 cells were seeded statically on either scaffolds (0.5 × 10^6^ cells/scaffold) or six-well plates (10^3^ and 2 × 10^3^ cells/cm^2^) and cultured for 10 days. At day 10, “primed” cells of each culture system were washed three times with PBS, trypsinized, and reseeded in well plates at the same cell density, namely, 30 × 10^3^ cells/cm^2^ on multiwell plates. After 2 additional days of culturing on 2D TCP, the culture medium and cells were collected to evaluate the expression of secreted and intracellular STCs, respectively.

### Cell Lysis and Protein Extraction

Cells were lysed in order to extract the total protein amount. The protein extract was further used in WB for the detection and comparison of protein expression levels of cells cultured under different conditions. Briefly, 10x RIPA (Radio Immuno Precipitation Assay) buffer (Cell Signaling, 9806, USA) was diluted in 1:10 in ddH_2_O and supplemented with a 1% v/v protease/phosphatase inhibitor cocktail (Fischer Scientific, 10137963, Rockford, USA). The adherent cells were washed on ice with precooled PBS for 15 min. Approximately 100 μL of RIPA was added per 0.3 × 10^6^ cells. Cells were incubated in cold buffer for 5 min. Cells in 2D substrates were scraped using cell scrapers, collected in sterile Eppendorf tubes, and centrifuged at 11000 rcf for 30 min at 4 °C. The pellets were discarded, and the supernatants were stored at −80 °C until further use. Scaffolds were collected in sterile Eppendorf tubes and immersed within the appropriate volume of buffer (based on the cell number attached) and incubated overnight at 4 °C. .After 1 day, after centrifugation at 11000 rcf for 30 min at 4 °C, the supernatant was collected and stored at −80 °C.

### Determination of Protein Concentration

Quantification of the total protein amount was achieved via colorimetric detection. A Bradford assay was performed using the bicinchoninic acid (BCA) kit (Thermo Scientific, 23227, Rockford, USA), following the manufacturer’s instructions. A BSA-based standard curve was plotted versus the absorbance at 562 nm with a microplate spectrophotometer (Thermo Scientific, Multiscan GO, 51119200, USA), and a linear regression fit with *R*^2^ > 0.99 was used to determine the total protein concentrations of samples.

### Western Blotting (WB)

After the total protein concentration of each sample was defined, the required sample volume corresponding to 15 μg of the total protein was used. A 4x Laemmli sample buffer (Bio-Rad, 161–0747) was added to the samples in 1:4 dilution and mixed (so that the final volume would be less than 40 μL). The proteins were denatured for 5 min at 95 °C using a thermomixer (Eppendorf, Germany) and centrifuged afterward, to collect any evaporation from tube walls. Precast gels (Bio-Rad Laboratories, 456-8094, USA) were placed in the electrophoresis tank. The space between gels was filled with 1× running buffer (0.302% w/v Tris base (Sigma-Aldrich, 201–064–4, St. Louis, MO) and 1.44% w/v glycine (Sigma-Aldrich, G8898, St. Louis, MO) in ddH_2_O). Prior to sample loading, wells were gently washed of any remaining acrylamide by pipetting 1 mL of 1× running buffer into them. Samples were loaded alongside with 4 μL of the Kaleidoscope protein ladder (Bio-Rad Laboratories, USA, 161-0375). Electrophoresis was run by increasing stepwise the initial voltage from 50 to 190 V (using a Bio-Rad Power Pac 1000). Proteins were separated on the basis of their size and molecular weight. After electrophoresis, separated proteins were transferred to PVDF membranes (Bio-Rad Laboratories, 170–4156, USA) using a transfer cassette (Bio-Rad Laboratories, Trans-Blot Turbo, USA). The protocol followed was for semidry transfer (25 V, 1A, 30 min). Negatively charged proteins moved up toward the positive cathode and onto the membrane. Thereafter, the membrane was transferred into a clean tray containing 25 mL of blocking solution (5% w/v milk or BSA in 1× TBS-T working buffer (2.75% v/v 5 M NaCl, 2% v/v 1 M Tris pH = 7.8, 0.1% v/v Tween-20 in ddH_2_O)) and incubated for 1 h at room temperature with gentle shaking. After membrane blocking, membranes were carefully cut in order to incubate each protein of interest with the respective primary antibody solution (in a 10 mL Falcon tube) overnight at 4 °C while being shaken. The primary antibodies polyclonal rabbit against human STC1 (1:1000, sc-30183, Santa Cruz Biotechnology, USA), polyclonal goat against human STC2 (1:1000, sc-14352, Santa Cruz Biotechnology, USA), polyclonal rabbit against human RANK (1:1000, sc-9072, Santa Cruz Biotechnology, USA), polyclonal rabbit against human Ki67 (1:1000, PA5–19462, Invitrogen, Schwerte, Germany), polyclonal rabbit against human α-tubulin (1:3000, ab126165, abcam, Cambridge, USA), polyclonal rabbit against human SOX2 (1:1000, AB5603, Chemicon International, Millipore, Billerica, MA, USA), polyclonal rabbit against human Oct3/4 (1:500, sc-9081, Santa Cruz Biotechnology, USA), monoclonal mouse against human GAPDH (1:4000, sc-365062, Santa Cruz Biotechnology, USA), monoclonal mouse against human lamin A/C (1:1000, ab9984, abcam, Cambridge, USA), and monoclonal mouse against human RANK (1:1000, ab12008, abcam, Cambridge, USA) were used. They were diluted in 2.5% w/v milk in 1× TBS-T buffer. After incubation, antibody solutions were discarded, and membranes were thoroughly washed three times in 1× TBS-T buffer. Each washing step lasted for 15 min. Washed membranes were incubated with horseradish peroxidase (HRP) conjugated goat or rabbit secondary antibodies against rabbit IgG (P0448), mouse IgG (P0447), or goat IgG (P0449), (Dako, Jena, Germany), depending on the primary antibody used, for 1 h at room temperature on a shaker. All secondary antibodies were diluted 1:4000 in 2.5% w/v milk in 1× TBS-T buffer.

After 1 h of incubation, antibody solutions were discarded followed by four washing steps in 1× TBS-T (each 15 min). Prior to imaging, membranes were incubated with an enhanced chemiluminescent (ECL) substrate (Thermo Scientific, 34095, USA) for 1 min and chemiluminescence imaging was performed using a CCD imager (ProteinSimpe, Fluor Chem Imager M, Westburg, The Netherlands).

After chemiluminescence and imaging, PVDF membranes were washed with 1× TBS-T and stored at 4 °C. The intensity of blots was semiquantified using the software ImageJ 1.47v. The intensity peak of the blot corresponding to the protein of interest was normalized to the respective peak of the loading control (housekeeping gene, here GAPDH or α-tubulin), resulting in the “relative band intensity”. This relative band intensity derived from each membrane was further normalized to the highest normalized band intensity observed in the specific experiment. In this way, the trends of different conditions per single membrane were kept, making feasible and reasonable the comparison and averaging of different experiments.

### Enzyme-Linked Immunosorbent Assay (ELISA)

ELISA assays were performed to quantify the levels of secreted STC1 and STC2 present in the conditioned cell culture medium under different culture conditions. Samples were centrifuged for 15 min at 4 °C at 1000 rcf, and their supernatants were assessed using the STC1 and STC2 ELISA kits provided by Cusabio (CSB-EL022821HU) and EIAAB (E12206h), respectively. Briefly, 96-well plates were precoated with the specific antibody against human STC1 or STC2. Standard curves were prepared from a dilution series of a standard (STC1 or STC2) concentration using the provided sample diluents, following the guidelines of the kits, and assayed alongside the unknown samples. In both kits, eight different dilutions were used for the subsequent plot of the standard curve. The detection ranges for STC1 and STC2 were 1.88–120 ng/mL and 31.2–2000 pg/mL, respectively.

Standards and samples were placed in each coated well. After 2 h of incubation at 37 °C the plate was further incubated for 1 h at 37 °C with a polyclonal biotin-conjugated antibody. This resulted in the formation of a complex with the antigen. Three washing steps with 1× wash buffer (mild detergent solution) for the removal of nonspecifically bound proteins were followed. Thereupon, horseradish peroxidase (HRP) conjugated avidin was added and the mixture incubated for 1 h at 37 °C. The wells were again thoroughly washed. Finally, a TMB substrate solution (90 μL/well) was added and an enzymatic reaction took place (in the wells where the protein of interest was present) during a 20 min incubation at 37 °C in the dark. The enzymatic reaction exhibited a change in color, indicating the quantity of antigen in each sample. The reactions were terminated by adding a sulfuric acid solution (50 μL/well). The color signal was measured spectrophotometrically at 450 nm with a microplate spectrophotometer (Thermo Scientific, Multiscan GO, 51119200, USA). The optical density was corrected by subtracting the reading at 540 nm. The concentration of the target protein in the corresponding samples was determined by using the standard curve (the polynomial regression line: protein concentration = *f* (absorbance) fit to the absorbance values of the standard samples with *R*^2^ ≥ 0.99). The secreted protein amount was determined (knowing the concentration from ELISA and the medium volume in which it was dissolved) and normalized to cell number. Technical triplicates were used for each biological replicate.

### Statistical Analysis

The statistical significance was determined by either a one-way ANOVA (Tukey’s posthoc test) using IBM SPSS statistics v22.0 or a Student’s *t* test using Microsoft Office Excel. Differences were significant (*) for *P* < 0.05 and highly significant (**) for *P* < 0.005. All experiments were performed at least twice, and in each experimental setup biological triplicates were used. Data are presented as averages; error bars indicate the standard deviation (SD).

## Results

### Determination of Surface Area of Fabricated Scaffolds

PA scaffolds with two different porosities were used in the present study. The different porosities arose from the different strand distances: that is, the horizontal distances between two successive parallel fibers measured from their centers. The actual strand distances were d2 = 465 ± 20 μm and d′2 = 650 ± 20 μm, as illustrated in Figure 1c,d, respectively. When it was taken into consideration that the diameter of one fiber was approximately d1 = 225 ± 20 μm, the space distances between two successive parallel fibers were c2 = 235 ± 15 μm and c′2 = 405 ± 10 μm, respectively. The layer thicknesses were the same in both cases: d3 = 145 ± 10 μm. Throughout the paper, scaffolds corresponding to strand distances of d2 = 465 ± 20 μm and d′2 = 650 ± 20 μm are named scaffolds with “small” pores (or low porosity) and “big” pores (or high porosity), respectively.

The available surface of the scaffolds (Table 1) was estimated using a mathematical model that took into consideration the macroscopic cylindrical shape of the scaffolds, the parameters d1, d2, and d3, the widening of the fibers at their junctions with the successive vertical layer, and the extraction of the unavailable part due to the overlapping of the filaments at their junctions (Appendix A in the Supporting Information). The computation of the surface area together with the cell numbers at different time points of cell culture allowed us to define the cell densities in the scaffolds and compare them to the respective densities on the 2D substrates.

**Table 1:** Available Surface Area of Scaffolds with Different Porosities^a^

scaffold	surface area (cm^2^)	surface area per unit volume Sν (mm^–1^)
small pores (d2 = 450 μm)	3.56	14.16
big pores (d2 = 650 μm)	2.76	11.06

a The corresponding surface area per unit volume Sv, defined as the ratio of surface per volume, is also demonstrated.

### Static Seeding Yielded a Sufficient and Homogeneous MDA-MB-231 Cell Distribution throughout the Scaffolds

COL-I coated scaffolds with big pores were used to evaluate the optimal cell seeding method in terms of cell attachment and distribution. Different cell densities were assessed: namely, 20 × 10^3^, 200 × 10^3^, 500 × 10^3^, and 1000 × 10^3^ cells/scaffold. After 1 and 7 day(s) of culture, methylene blue staining was performed to evaluate the cell distribution and attachment (Figure S1) for both methods of seeding. As depicted in Figure S1a, after 1 day of culture, dynamically seeded scaffolds appeared to be almost empty (no observable attached cells) under all different cell densities examined. At day 7 (Figure S1c), cell attachment was observed for just the two highest cell densities (500 × 10^3^ and 1,000 × 10^3^ cells/scaffold). In contrast, MDA-MB-231 cells attached successfully when they were seeded statically, with cell attachment being noticeable even after just 1 day of culture (Figure S1b), with a homogeneous cell distribution throughout the scaffolds. After 7 days of cell culture, methylene blue staining showed a higher cell density, implying cell proliferation within the scaffolds (Figure S1d). Additionally, no differences in cell distribution after 7 days of culture were observed on the top and bottom sides of the scaffolds. A similar cell attachment and distribution were obtained when scaffolds were coated with 20 μg/mL of FN instead of 100 μg/mL of COL-I (data not shown). Static seeding using COL-I coated scaffolds was used in the following experiments unless stated otherwise.

DAPI staining of cell nuclei was performed to assess the cell distribution along the scaffolds, which confirmed a homogeneous cell distribution along the different layers of scaffolds (Figure S2). Bright-field images confirmed cell attachment on the PA filaments (Figure S2), showing cells forming a monolayer on the fibers without aggregates. Moreover, closure of pores was not observed even after 10 days of culture on scaffolds with small pores. Conversely, mesenchymal stromal cells can fill the pore upon either seeding or prolonged cell culture, as previously reported.^33^

The seeding efficiency was assessed using the two different scaffold porosities. The seeding cell efficiency ranged between 7% for 3D scaffolds with big pores and 15% for scaffolds with small pores (Figure S3). Therefore, static seeding was chosen for the following experiments to compare the 3D culture systems to the conventional 2D TCP. The 2-fold higher seeding efficiency in the case of scaffolds with smaller pores can be partially attributed to the higher surface area (>29%) (Table 1).

### 3D Scaffolds Lead to Decreased Proliferation of MDA-MB-231 with Respect to 2D

To examine the cell proliferation and the expression of proliferation-associated markers in the different culture systems, MDA-MB-231 cells were cultured on 2D tissue culture plates (TCP) and on 3D PA scaffolds for 10 days.

**Figure 2:**
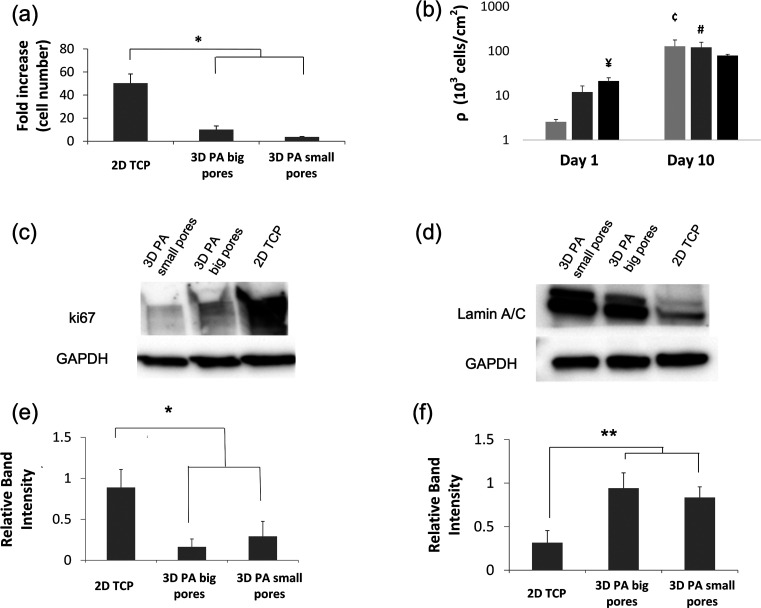
MDA-MB-231 cell proliferation and growth on 2D TCP and 3D PA scaffolds with big and small pores. (a) Fold increase in cell number after 10 days of cell culture in reference to the cell number at day 1. (b) Bar graph on a log scale showing cell densities determined at day 1 and day 10. ¥ denotes a significant difference from 2D TCP at day 1, ć denotes a highly significant difference from 2D TCP at day 1, and # denotes a significant difference from 3D big pores at day 1. Representative WBs for the detection and comparison of (c) ki67 and (d) lamin A/C among the different culture systems. GAPDH was used as a loading control. Semiquantitative analysis of ki67 (e) and (f) lamin A/C expression based on WB confirming that the intensity of the ki67 blot is much higher in the 2D culture, whereas the opposite tendency is observed in the case of lamin A/C. Biological triplicates were used in each experiment. Error bars represent SD; * and ** denote statistically and highly statistically significant differences, respectively (*P* < 0.05 and *P* < 0.005).

MDA-MB-231 cell proliferation was markedly higher on the 2D TCP in comparison to both scaffolds of different porosities (Figure 2a). More specifically, the cell number was amplified 50 times in 2D plates, whereas only 10 and 4 times in higher and lower porosity 3D scaffolds, respectively.

The increased proliferation of cells cultured on 2D (in comparison to 3D) was confirmed by evaluating the cellular expression levels of proliferation-associated markers. A positive correlation between ki67 expression and cell proliferation was observed; whereas, the opposite trend was found between lamin A/C expression and cell proliferation. ki67 (Figure 2c,e) was more expressed in the 2D culture than in 3D scaffolds. In contrast, the lamin A/C blot intensity was lower in the case of the 2D culture (Figure 2d,f).

### MDA-MB-231 Stemness Is Not Affected by the Dimensionality of the Culture System

**Figure 3:**
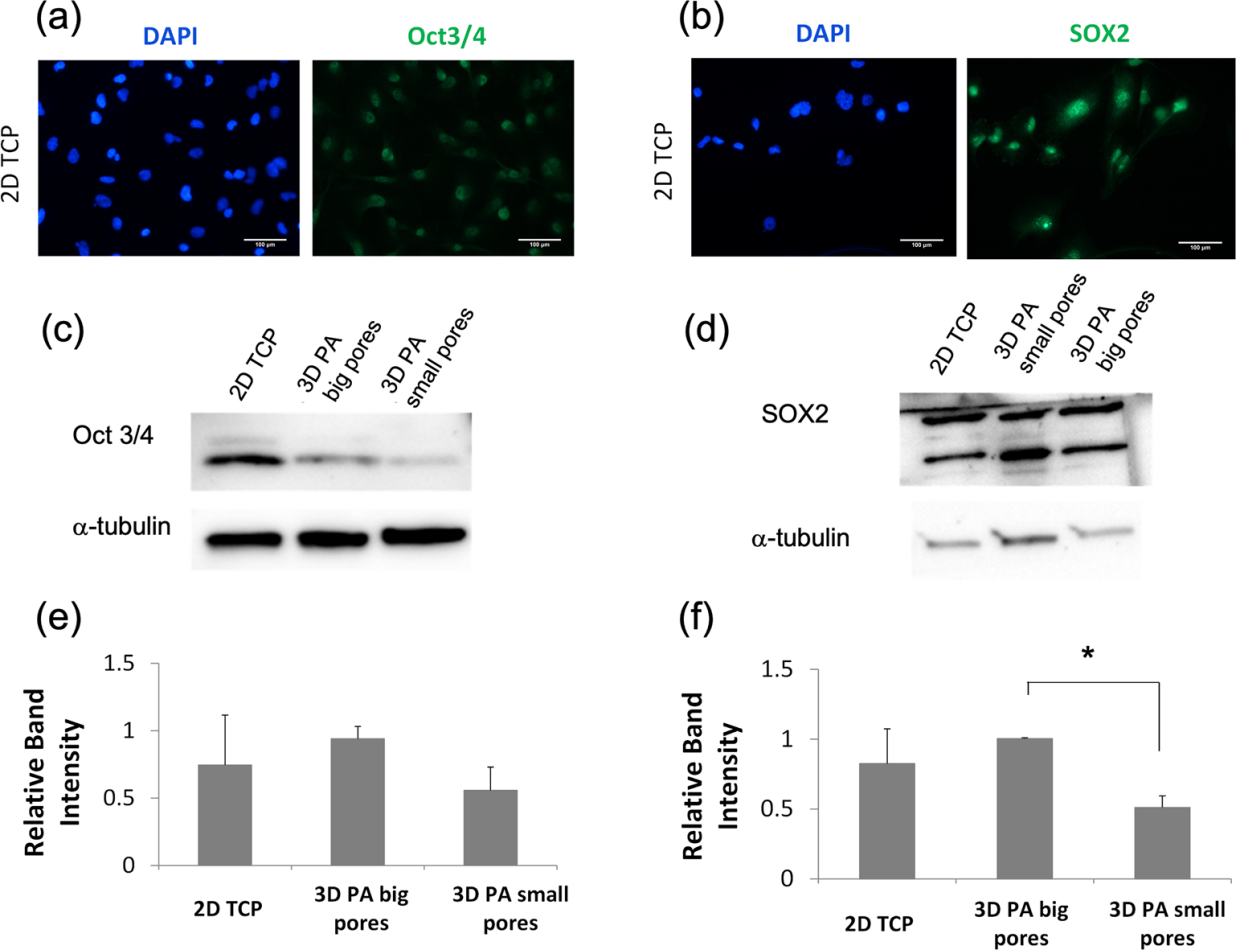
Expression of stemness-related transcription factors in MDA-MB-231 cells. (a) Immunofluorescence staining and imaging of (a) Oct3/4 and (b) SOX2 expressed in MDA-MB-231 cells cultured on 2D TCP. DAPI staining (in blue) and Oct3/4 and SOX2 (in green) showed that Oct3/4 and SOX2 are localized mainly within cell nuclei. Scale bars represent 100 μm. WBs for a comparison of (c) Oct3/4 and (d) and SOX2 expression levels among the different culture systems. α-Tubulin was used as a loading control. Relative intensities of (e) Oct3/4 and (f) SOX2 blots to α-tubulin are not significantly different between 2D and 3D cultures. Error bars represent SD, and * denotes a statistically significant difference (*P* < 0.05).

To evaluate whether the 3D culture dimensionality affected the stemness of cancer cells, we examined the expression levels of the two stemness-related transcription factors: namely, Oct3/4 and SOX2. The expression of the aforementioned proteins was first confirmed by IF (Figure 3a,b) in 2D cultures. A comparison of expression levels among the different culture systems was assessed by WB (Figure 3c,d).

A semiquantitative analysis of different blots revealed no significant changes for Oct3/4 (Figure 3c,e), whereas a significant decrease of normalized SOX2 levels was seen only when MDA-MB-231 cells were cultured in 3D scaffolds with lower porosity in comparison to the respective values of 3D scaffolds with higher porosity (Figure 3d,f). No significant differences in SOX2 levels were noted between 2D and 3D cultures.

### Dormancy of MDA-MB231 Significantly Increased on 3D Scaffolds

To evaluate whether the 3D culture conformation influenced cancer cell dormancy, the intracellular expression levels of STC1 and the secreted levels of both STC1 and STC2 of MDA-MB-231 cells cultured in the different configurations were compared after 8 and 10 days of culture, respectively. To further elucidate whereas other factors influence the secretion of STCs, namely, the material out of which the 3D scaffolds were fabricated and the cell density, we included 2D TCP cultures of two different densities at day 0, specifically ρ = 10^3^ and 2ρ = 2 × 10^3^ cells/cm^2^, as well as PA disks (2D cell culture conformation made of PA) seeded at 2ρ = 2 × 10^3^ cells/cm^2^.

**Figure 4:**
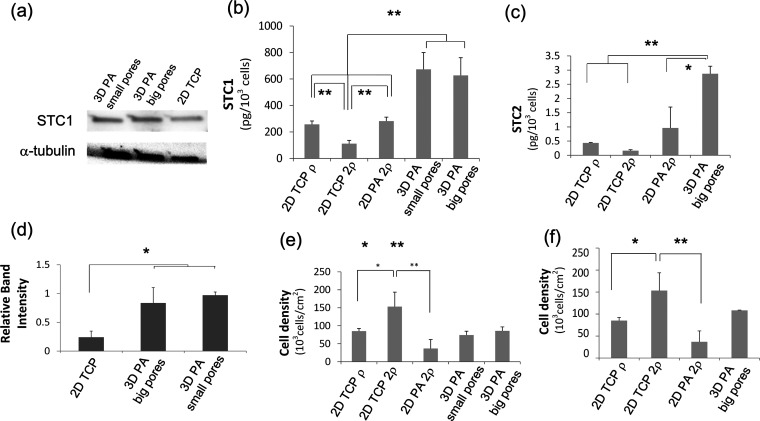
Expression of dormancy-related markers in MDA-MB-231 cells cultured in different configurations. (a) WB corresponding to the intracellular STC1 expression of MDA-MB-231 cells cultured for 8 days on either 2D TCP or 3D PA scaffolds. α-Tubulin was used as a loading control. (b) Bar graph corresponding to normalized to cell number STC1 secretion, measured by ELISA from the supernatant of cell cultures at *t* = 10 days. (c) Bar graph corresponding to normalized to cell number STC2 secretion measured by ELISA from the supernatant of cell cultures at *t* = 10 days. (d) Bar graph representing WB semiquantitative analysis of STC1 expression corresponding to normalized (to α-tubulin) STC1 expression after 8 days of cell culture on the various systems. (e) Average cell density corresponding to each culture condition of STC1 experiments. The cell number of each scaffold (data not shown) was used for the STC1 normalization corresponding to each scaffold. (f) Average cell density corresponding to each culture condition of STC2 experiments. The cell number of each scaffold was used for the STC2 normalization of each scaffold. Biological triplicates were used per experiment, and technical triplicates were used during the performance. Error bars represent SD, and * and ** denote *P* < 0.05 and *P* < 0.005, respectively.

Both 3D PA scaffold cell culture systems resulted in significantly increased intracellular STC1 expression levels at day 8 in comparison to the respective levels of 2D cultured cells (Figure 4a,c).

Secreted STC1 and STC2 levels evaluated by ELISAs were also significantly higher when cells were cultured on 3D scaffolds in comparison to all of the different 2D cultures in a porosity-independent manner (Figure 4b,c). As all 2D conditions tested (cell density and biomaterial) showed reduced secretion, dimensionality is the main factor for STC1 and STC2 secretion.

However, cell density impairs cell dormancy. Cells with significantly higher cell densities (initial 2ρ) (Figure 4b,e) secreted significantly lower STC1. STC2 release was lower in higher cell densities but was not significantly different (Figure 4c,f).

The STC1 release from 2D TCP (2ρ) was significantly decreased in comparison to the 2D PA culture of the same cell density (2ρ); however, cells on PA disks grew at lower rates, as indicated by the significantly lower final cell density on 2D PA.

Consequently, our data corroborated a different behavior of cells with altered spatial arrangements. In the case of the 2D culture, an increase in cell density resulted in decreased STC1 and STC2 release levels. Nevertheless, cell dormancy was clearly increased on 3D scaffolds.

### Plasticity of MDA-MB-231 Cells

**Figure 5:**
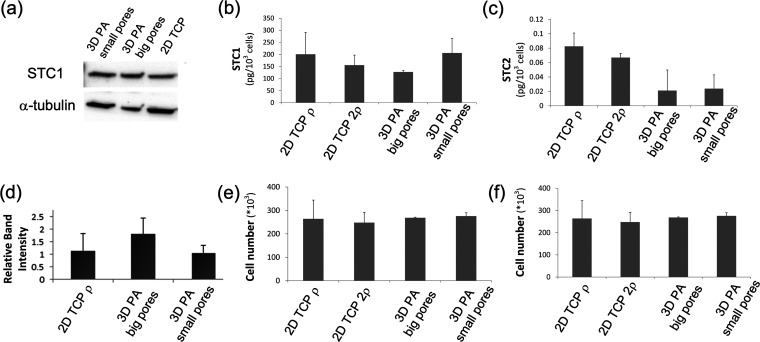
Cell plasticity of MDA-MB-231 cells precultured in different cell culture configurations. (a) WB corresponding to the intracellular STC1 expression of “primed” MDA-MB-231 cells further cultured for 2 days on TCP under the same cell densities. α-Tubulin was used as a loading control. (b) Bar graph corresponding to secreted STC1 normalized to cell number. STC1 is measured by ELISA from the supernatant of “primed” MDA-MB-231 cells after 2 days of culture on TCP under the same cell densities and normalized to the cell number shown in the (e) bar graph. (c) Bar graph corresponding to STC2 secretion normalized to cell number. STC2 is measured by ELISA from the supernatant of “primed” MDA-MB-231 cells after 2 days of culture on TCP under the same cell densities and normalized to the cell number shown in the (f) bar graph. (d) Bar graph representing WB semiquantitative analysis of STC1 expression corresponding to STC1 expression after 2 days of cell culture and normalized to α-tubulin. (e) Average cell number determined by DNA quantification corresponding to each culture condition of STC1 experiments. (f) Average cell number determined by DNA quantification corresponding to each culture condition of STC2 experiments. Biological triplicates were used per experiment, and technical triplicates were used during the performance of ELISAs. Error bars represent SD. One-way ANOVA shows no statistical differences among all groups of “primed” cells once cultured for 2 additional days on 2D TCP. One-way ANOVA shows no statistical differences of cell number among all groups in the bar graph (e) corresponding to STC1 secretion experiments and the bar graph (f) corresponding to STC2 secretion experiments.

An evaluation of the robustness of the model and the plasticity of MDA-MB-231 cells was also assessed by removing the cells from a 3D environment after 10 days and reseeding in a 2D microenvironment for additional 2 days (priming experiments) (Figure 5).

Normalized STC2 release appeared to be lower when cells were precultured on scaffolds. However, one-way ANOVA indicates that there is no significant difference on secreted STC2. In addition, there is no statistical difference in normalized secreted STC1. Also, a statistical analysis showed no significant differences of cell number among all groups in any of the experiments corresponding to STC1 and STC2 secretion.

### ECM-Derived Cells

**Figure 6:**
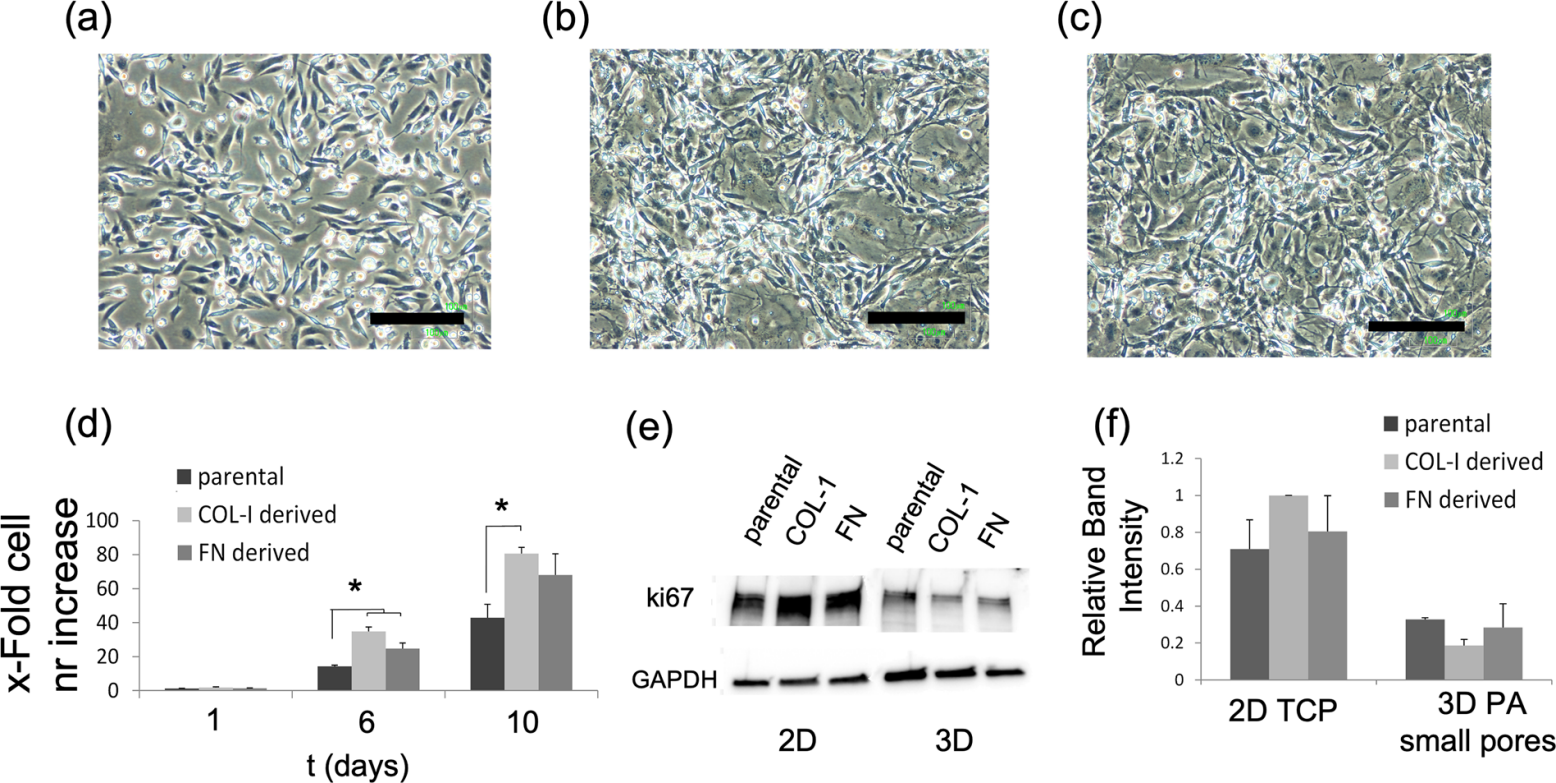
Characterization of ECM-derived MDA-MB-231 cells. Bright-field images showing the morphology of (a) parental, (b) COL-I-derived and (c) FN-derived MDA-MB-231 cells, cultured on 2D TCP. Black scale bars represent 200 μm. Filial cells exhibit a more mesenchymal morphology in comparison to the parental MDA-MB-231 population. (d) Proliferation rate on 2D TCP as shown by the fold cell number increase of parental and COL-I and FN-derived MDA-MB-231 cells. Cell numbers were determined at 1, 6, and 10 days. (e) WB of ki67 for parental and COL-I and FN-derived MDA-MB-231 cells under 2D TCP and 3D PA (of smaller porosity) cultures. The total protein amount was extracted at 6 and 8 days in the case of 2D and 3D cultures, respectively. GAPDH was used as a loading control. (f) Normalized (to GAPDH) ki67 expression based on band intensity peaks in the case of 2D and 3D cultures, respectively. Normalized ki67 levels of 2D and 3D correspond to 6 and 8 days, respectively. Biological triplicates were used per time point. Error bars represent SD. A statistical analysis among different cell lines was carried out with one-way ANOVA. * denotes *P* < 0.05.

Morphology, proliferation, and RANK expression were studied in parental MDA-MB-231 and cells derived on COL-1 and FN extracellular matrices (ECM). COL-I- and FN-derived MDA-MB-231 cells appeared to be more elongated and with a “mesenchymal-like” shape in comparison to the parental population (Figure 6a–c). Filial cells showed an increased tendency to grow in colonies with respect to the parental cells. Furthermore, COL-I (Figure 6b) filial cells were arranged in a more sophisticated manner, forming ring structures within which void spaces were observed. In contrast, parental cells were more homogeneously distributed when they were cultured, without distinct cell conformations being observed (Figure 6 a).

As ECM-derived cells develop an increased growth rate, we assessed the growth rate of parental and filial cell lines by culturing them on TCP plates with a starting cell density of 5 × 10^3^ cells/cm^2^. Plates were not additionally coated with either COL-I nor FN. The cell number was determined at 1, 6, and 10 days. At day 1, no significant differences were observed among different cell lines. However, at 6 days both COL-I- and FN-derived cells displayed a significantly higher rate in comparison to the respective parental cells. Similarly, at 10 days the growth rate of COL-I-derived cells was significantly higher relative to that of parental cells (Figure 6 d).

ki67 intracellular expression levels in 2D in the ECM-derived MDA-MB-231 confirms the results found previously for parental cells. ki67 intracellular expression levels were compared among parental and filial cell lines on both 2D and 3D cultures by performing WB (Figure 6e). Normalized ki67 values after WB semiquantification (Figure 6f) revealed similar ki67 expression levels on the 2D culture. ki67 levels were more highly expressed in COL-I-derived cells, followed by FN-derived and parental cell. However, a different trend was observed in the case of 3D cultures, albeit it was not significantly different.

### RANK Expression Is Associated with ECM Cell Affinity

**Figure 7:**
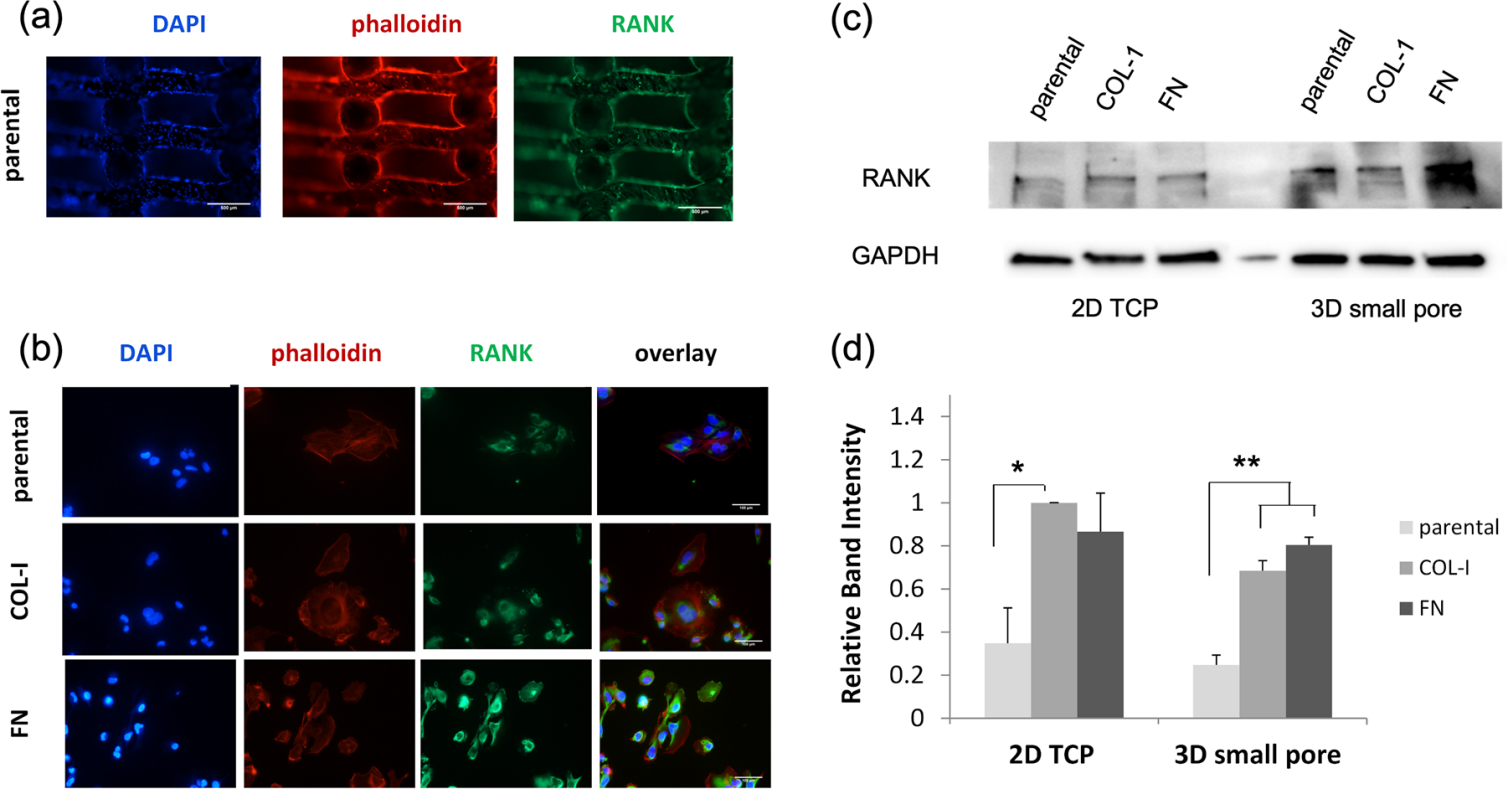
Cell morphology and RANK localization of parental and filial MDA-MB-231 cells on (a) a 3D PA scaffold and (b) a 2D TCP. Fluorescent staining: nucleus (blue), actin filaments (red), and RANK protein (green). Scale bars represent 500 and 100 μm for 3D and 2D cultures, respectively. (c) WB of RANK for parental and COL-I- and FN-derived MDA-MB-231 cells under 2D and 3D (of small pores/lower porosity) cultures. The total protein amount was extracted after 3 and 8 days in the case of 2D and 3D cultures, respectively. GAPDH was used as a loading control. (d) Normalized (to GAPDH) RANK expression based on the intensity peaks of the respective Western blots. Error bars represent SD, and * and ** indicate significant and highly significant statistical differences, respectively.

We next examined if an association between the expression profiles of RANK receptor and the cell affinity to ECM proteins, namely, COL-I and FN, could be found. Parental and COL-I-and FN-derived MDA-MB-231 cells were cultured on 2D plates as well as 3D scaffolds. In the latter case, we used only scaffolds with small pore size due to their potential for increased cell seeding efficiency with respect to scaffolds with larger pores (Figure S3). On the basis of the IF images obtained from 2D cultures (Figure 7b), COL-I-derived cells appeared to be more round in comparison to the parental population, which displayed a more ellipsoid shape. FN-derived cells were more elongated in relation to parental cells, with their elongation being even more noticeable with increasing cell confluence. RANK was also visualized when cells were cultured on 3D PA scaffolds (Figure 7a).

WB for the detection and comparison of expression levels of RANK among parental and filial MDA-MB-231 cells in 2D and 3D cultures was performed (Figure 7c). A semiquantitative analysis of blots suggested significantly elevated levels of RANK by COL-derived cells in comparison to parental cells when they were cultured on 2D; no significant difference was found between COL-1- and FN-derived cells. When cells were cultured in 3D PA scaffolds, the difference in expression levels of RANK became more profound between derivative cell lines and parental cells. In particular, RANK was highly increased in both filial cell lines in comparison to the respective expression of the parental population (Figure 7d).

## Discussion

In the field of tissue engineering, AM is a transformative technology based on layer by layer deposition of a material for the fabrication of 3D scaffolds with complex designs. Although the technique is now a recognized procedure, it allows the creation of new opportunities for manipulating and mimicking the intrinsically multiscale and multifunctional structures in the *in vivo* microenvironment. The goal presented in this paper is to generate a 3D *in vitro* model, which shows that cancer cells on printed scaffolds can be used in the search for new predictive markers and new targets for anticancer treatment. The model presented provides a high interfacial bond area, ideal for vascularization and bone ingrowth. Adding another dimension of complexity by reducing the pore size allowed us to further target the design of the 3D scaffold for the control of cell growth and support for cell adhesion. Moreover, as 3D models for *in vitro* studies are increasingly becoming in high demand in cancer research, the differences between 2D and 3D models were assessed in order to uncover the complexity of the tumor.

Cell seeding and distribution throughout a scaffold is a key parameter, as it has been shown to influence the proliferation, migration, and differentiation of cells. Optimization of the seeding process is crucial, especially to preserve cell viability and to provide a spatially uniform distribution as well as to minimize the donor site morbidity when donor cells are collected from biopsies. Thus, the seeding technique must allow a uniform distribution and adherence of cells throughout the scaffold. Previous techniques have involved static^34^ and dynamic^35−37^ seeding. Dynamic techniques such as flow perfusion, centrifugation, orbital shaking, and a spinner flask are still limited within AM scaffolds due to their intrinsic characteristics, such as large and interconnected pore architectures or the lack of specific cell adhesion sites.^38−40^ Moreover, some dynamic seeding techniques are complex and necessitate specific equipment and expertise, showing a decreased cell number in seeding in AM 3D scaffolds.^41^ One aim of this work was to compare both dynamic and static seeding for 3D scaffolds of different porosities. We showed that efficient and homogeneous cell attachment along the 3D scaffolds was achieved only with static seeding, as previously reported.^27^

A reduction in cell proliferation in 3D cultures has already been observed in several cell lines in comparison to 2D cultures.^42,43^ For example, in the study conducted by Adcock and co-workers,^42^ DU145 cells cultured in 3D showed a lower proliferation rate in comparison to their counterpart in a 2D culture. A 2D monolayer cell culture allows a higher cell proliferation rate due to the different amount of nutrients and growth factors from the culture medium and to the higher prevalence of proliferative cells with respect to necrotic cells. However, a 3D structure allows cell growth at different stages, such as proliferation, quiescent, apoptotic, hypoxic, and necrotic, due to the limited diffusion of culture medium to the cells.^44^ In fact, Imamura’s group^45^ reported a 3D breast cell culture that mimicked important tumor characteristics, such as hypoxia, dormancy, and antiapoptotic features, in comparison to a 2D culture. In our work we observed a reduced cell proliferation in 3D AM scaffolds and an increased dormant phenotype and lamin A/C expression and overexpression of STCs. Moreover, different cell viabilities in 3D may be related to the absence of oxygen in the cellular environment, which also triggers an increased cellular population in the G0 phase.^46^ We assessed cell proliferation in the different culture systems through expression levels of the proliferation marker ki67, which is present in all cell nuclei of all cell cycle phases but not in the quiescent or the G0 phase. As expected, ki67 was significantly overexpressed in 2D in comparison to 3D PA scaffolds of both porosities.

Biophysical cues can trigger the epithelial–mesenchymal transition (EMT) with loss of cell–cell adhesion, changes in geometry, and an augmented matrix elasticity.^47,48^ For instance, Greenburg and Hay reported that varying the mechanical and geometric properties of the environment triggered EMT in epithelia seeded on 3D matrices.^48^ Moreover, recent reports determined that overexpression or knockdown of lamin A/C can affect EMT biomarkers, suggesting that lamin A/C might be central in the occurrence of EMT.^49^ Substrate’s dimensionality has also been linked to lamin A/C expression.^50^ In fact, human mesenchymal stromal cells (hMSCs) cultured on 3D electrospun scaffolds showed fewer focal adhesions and a lower lamin A/C expression in comparison to 2D substrates.^50^ Roncato reported that downregulation of lamin A/C in melanoma and breast carcinoma did not affect cell proliferation in 2D substrates but impaired it in 3D spheroids within soft agar.^51^ Our findings showed an increase in lamin A/C in 3D substrates. Certainly, experiments on the invasive behavior and with lamin and EMT markers are required before definitive conclusions can be drawn. However, we can assume that our findings indicated that the expression of lamin A/C can be regulated by the cell microenvironment. Type A lamins have been inversely correlated with increased proliferation rates. The increased proliferation rates in 2D cultures could result in the lack of lamins due to the fast nuclear envelope assembly/disassembly and, consequently, the underdevelopment of the required nuclear structure. On the other hand, the lower proliferation rates observed in cells cultured in 3D scaffolds allow them to follow all the necessary steps so that the structure of nuclear lamina at the periphery and through the nucleoplasm can be developed. A correlation between the increased cell proliferation and the reduced or absent Lamin A expression, consistent with our data, has been previously described.^52^

Although overexpression of lamin A/C has been related to increased aggressiveness and motility and a poor prognosis in human prostate, ovarian, and colon cancers,^53,54^ in the case of breast cancer, it is associated with less aggressive tumors.^55^ Venables and co-workers proposed that cancer cells lost their differentiated phenotype, producing lower levels of lamin A.^52^ Our results suggested that MDA-MB-231 cultured on 3D scaffolds could retain their differentiated phenotype for a longer time in comparison to the respective cells cultured on conventional Petri dishes, implying that 3D scaffolds may represent an improved culture model to study cancer cells isolated from cancer patients (for example circulating tumor cells or tumor cells isolated from needle biopsies) as well as to serve as a drug screening platform.

The stemness of cancer cells on 3D and 2D cultures was addressed by assessing the expression levels of the embryonic transcription factors SOX2 and Oct3/4, known to regulate embryogenesis and maintain the pluripotency and self-renewal capacity of stem cells.^16^ Studies have shown that genes, whose expression is constrained to undifferentiated or proliferative cells, are wrongly overexpressed in breast cancers, whereas others, expressed normally in mammary gland, are lost.^56^ In our study, no significant differences were found in the expression levels of SOX2 and Oct3/4 between the 2D and 3D cultured cells. It is generally accepted that 3D models maintain the pluripotency and self-renewal capacity of stem cells to a much higher degree in comparison to conventional 2D cultures.^57^ In accordance, many studies have also suggested an enhanced stemness of cancer cells when they are cultured on 3D scaffolds.^58^

Cancer cell dormancy is a process whereby cells enter in a reversible cell cycle arrest, also called quiescence. The dormancy of cells cultured in the different cell culture configurations was addressed by assessing their STC1 and STC2 intracellular and secretion levels by Western blotting and ELISA, respectively. Our results showed significantly increased secretion of STC1 and STC2 from cells cultured in 3D in comparison to 2D in a porosity-independent manner. Intracellular STC1 expression was consistent with the ELISA data with regard to the tendencies observed. After including some additional controls (2D PA disks and different cell seeding densities), we concluded that the most reliable parameter behind the dormant cell phenotype is the dimensionality, resulting in a 3D spatial cell arrangement. A previous study carried out by Hou and colleagues indicated that, in human breast cancer cells, STC2 may inhibit EMT through the activation of a protein kinase C (PKC) signaling pathway.^59^ However, in another study, abnormal STC-1/2 expression has been correlated with tumorigenesis and poor clinical outcomes in ovarian and colorectal cancers.^60^ Although the clinical significance of STC-1 expression and the intracellular signaling events underlying the response of STCs to their microenvironments in breast cancer remain elusive, our findings indicated that the expression of STC1 and secreted levels can be influenced by the cell microenvironment.

The chemistry of PA and its effect on the reduced cell growth observed on both disks and scaffolds are interrelated. In fact, PA contains α-tocopherol, a synthetic form of vitamin E serving as an antioxidant,^61^ which does not lead to cancer cell death.^62^ Together, our results suggested that PA scaffolds promote cancer cell dormancy, with significantly decreasing proliferation rates. Quiescent cells are more resistant to standard chemo- and radiotherapies due to their slow cycling potential. Additionally, quiescence is essential for cancer cells to acquire additional mutations, to survive in a new environment and initiate metastasis, to become resistant to cancer therapy, and to evade immune destruction.^63^

Cell affinity to different ECM proteins, such as COL-I and FN, has been involved in metastatic processes.^64^ In addition, RANK has been found to be a promising prognostic^65^ and predictive marker to denosumab treatment response in breast cancer.^66^ A previous report from Park and Helfman^67^ showed an upregulation of FN in MDA-MB-231 cells when cultured in 3D suspension. The increased FN expression of 3D cultured cells promoted their metastasis while in circulation via enhanced attachment to secondary organs. Herein, we investigated a potential relationship between ECM and RANK protein expression by comparing the upregulation of RANK protein levels of parental cells as opposed to ECM-derived cells.^29^ The ECM-derived cell lines exposed differences in morphology, proliferation, and RANK levels in comparison with the parental MDA cell line. The filial MDA-MB-231 cells exhibited a more mesenchymal morphology which is associated with EMT and higher RANK levels in comparison to the parental cells. Our results are supported by the findings of Palafox and co-workers, who reported that increased RANK/RANKL levels were related to increased EMT.^68^ COL-I- and FN-derived cells proliferated with significantly higher rates when they were cultured on 2D in comparison to the parental MDA-MB-231 cell population. This can be attributed to the integrin binding to ligands in the ECM, which enhances cell attachment, initiating several prosurvival mechanisms to prevent apoptosis.^69^ Moreover, it has long been appreciated that attachment to protein substrates reinforces cell growth.^70^

All in all, our results suggested that RANK expression is associated with the cell affinity to different ECM proteins. It remains to be demonstrated in prospective studies whether cancer patients with RANK+ tumors can benefit from treatments blocking the integrins associated with the affinity to different ECM proteins by preventing bone metastasis and disease progression.

## Conclusion

Porous 3D PA scaffolds were fabricated by additive manufacturing. MDA-MB-231 breast cancer cells were used due to their high metastatic potential. 3D cultures were optimized for cell seeding and attachment, aiming for a system containing sufficient, evenly distributed cells and comparable cell densities among three cell culture systems: namely, (i) 2D TCP, (ii) 3D PA scaffolds with big pores, and (iii) 3D PA scaffolds with small pores.

The protein expression profile involved in proliferation, dormancy, and stemness was assessed. MDA-MB-231 cells cultured on 3D scaffolds and 2D flat surfaces adapted differently to their environment. 3D environments led to increased STC1, STC2, and lamin A/C expressions. The altered behaviors of STC1 and STC2 expression levels of cells were ascribed to both the chemistry of the polymer used and the dimensionality of the culture model. Metastatic MDA-MB-231 cells in this *in vitro* 3D cancer model become more dormant and are hence a relevant cell phenotype for the development of anticancer drugs with more predictive clinical outcomes.

ECM-derived MDA-MB-231 from COL-I and FN exhibited a more mesenchymal-like morphology and proliferated significantly more highly than the parental population. The expression levels of RANK in COL-I- and FN-derived cells were significantly higher in both 2D and 3D cultures in comparison to the parental cells. Osteotropism of cancer cells correlates with the mesenchymal-like phenotype and RANK expression; therefore, derivative cell lines could become more bone metastatic *in vivo*.

This work may contribute to engineering relevant cell culture systems that better recapitulate human pathophysiology and cancer hallmarks *in vitro*.

## Supporting Information Available

Mathematical determination of the available scaffold surface area, images of methylene blue stained scaffolds (with big pores) seeded dynamically and statically, cell distribution on 3D scaffolds, and efficiency of static seeding on scaffolds of different porosities (PDF)

## ABBREVIATIONS

TCP: tissue culture polystyrene
STC1: stanniocalcin 1
STC2: stanniocalcin 2
ECM: extracellular matrix
COL-I: collagen type I
FN: fibronectin
PA: polyactive
WB: Western blotting
ELISA: enzyme-linked immunosorbent assay
RANK: receptor activator of NFκB
PEOT/PBT: copolymer poly(ethylene oxide terephthalate)/poly(butylene terephthalate)
