# Supplementary material for: 3D Culture Modeling of Metastatic Breast Cancer Cells in Additive Manufactured Scaffolds
Source: ACS Appl Mater Interfaces. 2022 Jun 10;14(24):28389–402. doi: 10.1021/acsami.2c07492 (PMC9227707; doi:10.1021/acsami.2c07492)
Supplement: Supplementary file 1 — am2c07492_si_001.pdf [file am2c07492_si_001.pdf]

Supporting Information

3D culture modelling of metastatic breast cancer cells in additive manufactured scaffolds

Afroditi Nanou[1,2]*, Ivan Lorenzo-Moldero*[1,3]*, Kyriakos D. Gazouleas*[1]*, Barbara Cortese**[4]*, Lorenzo Moroni**[1,3,4]

[1]Tissue Regeneration department, MIRA Institute for Biomedical Technology, University of Twente, Drienerlolaan 5, Enschede, The Netherlands

[2]Medical Cell BioPhysics department, Faculty of Science and Technology, University of Twente, Dienstweg 1, 7522 ND, Enschede, The Netherlands

[3]Complex Tissue Regeneration department, MERLN Institute for Technology-Inspired Regenerative Medicine, Maastricht University, Universiteitssingel 40, 6229 ER Maastricht, The Netherlands.

[4] National Research Council-Nanotechnology Institute (CNR Nanotec), 00185 Rome, Italy.

*Corresponding Authors: Email: barbara.corese@nanotec.cnr.it; l.moroni@maastrichtuniversity.nl

Appendix A: Determination of the available scaffold surface area

A mathematical model to determine the available surface area of a scaffold was developed, Scheme 1. The steps below were followed:

Determination of the surface of a cubic block tangent to the cylindrical scaffold (orange square).

Determination of the lower bound of the scaffold area (green polygon).

Determination of the upper bound of the scaffold area (red polygon).

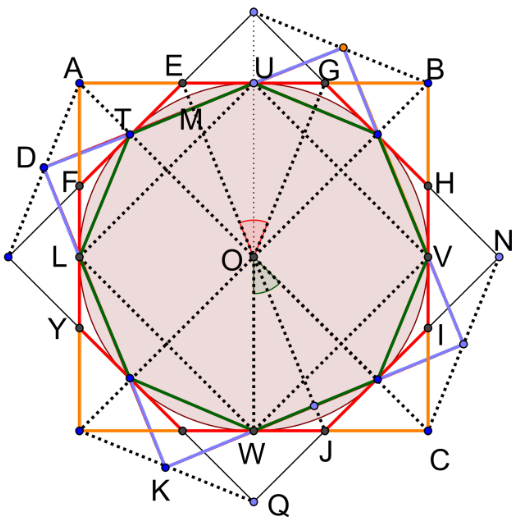

Scheme 1: Scheme illustrating the top view of the macroscopic cylindrical scaffold (purple area). The orange square is tangent to the cylindrical scaffold, the red and green polygons represent, in the resent model, the upper and lower bound respectively.

Determination of the surface of a cubic block tangent to the cylindrical scaffold of d=4mm (indicated with orange).

Measurement of the amount of fibers per layer

$$d_2 \cdot k + (k\text{-}1) \cdot c = d \Rightarrow k = \frac{d + c}{d_2 + c}$$

where:

k the number of fibers

d the length and width of the square block (d=diameter of cylindrical scaffold)

c the (void) distance between 2 fibers

d_2 the fiber diameter (250 μm)

We are interested in the amount of the fiber parts (between two junctions or between a junction and an edge of fiber) created per pair of layers, which will be equal to

$$\underbrace{3+(k-2)\cdot 2+3+(k-2)\cdot 2+.....+3+(k-2)\cdot 2}_{(k-1)\,\text{times}}$$

We should add to that the number of fiber parts of the last column, which is $k-1$.

Hence, in total:

$(k-1)\cdot[3+2\cdot(k-2)]+k-1=2k^2-2k$ fiber parts are created in each pair of layers.

For v pairs, it will be $v(2k^2-2k)$

The total area of these cylinder-like fiber parts will be $v(2k^2-2k)250\pi(c-20)=500\pi(c-20)v(k^2-k)$,

where ($c-20$) derives from the height of these fibers that will be reduced by approximately 8% of the fiber diameter due to the fiber widening at the joining points

$4\%\cdot 250\mu m=10\mu m$,

Therefore, from the initial fiber distance C this distance should be abstracted twice (from both sides).

If the block consists of odd number of layers, then we should add also the cylinders of the upper layer, which are $k^2 - k$ in number and their surface equals to $(k^2 - k)250\pi$ c

Then, their total surface area will be

$$500\pi(c-20)v(k^2-k)+(k^2-k)250\pi \text{c}$$

We should, however, take also into account the available area of the junctions. Only the junctions of the upper and the lower layer contribute to the final available surface area. These junctions will have an area equal to the square ABCD (Scheme 2) subtracted by the 4 (equal area) circular sections with each of them having an area equal to FAE.

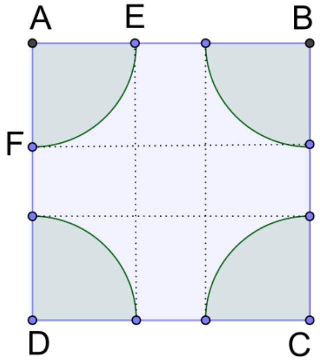

Scheme 1

The side of the square is defined as $AB = 250 + \frac{8}{100}250 = 270\mu m$ and the radius of each circular section is $\frac{270-250}{2} = 10\mu m$

Hence, $E_{FAE} = \frac{\pi 10^2 90}{360} = 25\pi$.

Therefore, the area in each joining point will be $E_{junction} = 270^2 - 4 \cdot 25\pi$

The total number of junctions is $2k^2$ (of the top and bottom layer) with a total surface area of $2k^2 E_{junction} = (270^2 - 4 \cdot 25\pi)2k^2$

Furthermore, in the 4 external sides of every pair of layers, we have $4k$ ellipses derived from the fibers spreading at the joining points (until they solidify). These ellipses have totally a surface $4k\pi \frac{270}{2} \cdot \frac{150}{2} = 40500k\pi$, corresponding to 1 pair of layers. For the whole block we should multiply by ν. So, we will have the surface $40500k\pi\nu$

If, though, the number of layers is not even, then we will have $2k$ more ellipses. So, the total area will be slightly different and equal to $40500k\pi\nu + 2k\pi \frac{270}{2}\frac{150}{2} = 40500k\pi\nu + k\pi 20250$

Finally, the cylindrical surfaces at the internal sides of the joints should be also added, since they contribute to the final available surface. For each juncture, there are two surfaces (in the front and in the back).

In case of even number of layers, the number of these surfaces will be $4k^2(\nu - 1)$ with their area being equal to $4k^2(\nu - 1)\frac{E_{cylinder,junction}}{2} = 2k^2(\nu - 1) \cdot E_{cylinder,junction}$

In case of odd number of layers, these surfaces will be in multitude of $2(\mathrm{v}-1)\mathrm{k}^2 + 2\nu k^2 = k^2(4\nu - 2)$ with their total area

$$k^2\,(4\nu - 2)\frac{E_{cylinder,junction}}{2} = k^2(4\nu - 2) \cdot E_{cylinder,junction}$$

In summary, the total available surface area of the block for even number of layers will be:

$$\mathrm{E} = \nu\left[(2\mathrm{k}^2\text{-}2\mathrm{k}) \cdot \mathrm{E}_{\mathrm{cylinder}} + 4\mathrm{k} \cdot \mathrm{E}_{\mathrm{ellipsis}}\right] + 2\mathrm{k}^2 \cdot \mathrm{E}_{\mathrm{junction}} + 4\mathrm{k}^2(\nu\text{-}1)\frac{\mathrm{E}_{\mathrm{cylinder\cdot\,junction}}}{2}$$

$$= 2\nu(\mathrm{k}^2\text{-}\mathrm{k})\,\mathrm{E}_{\mathrm{cylinder}} + 4\mathrm{k}\nu\mathrm{E}_{\mathrm{ellipsis}} + 2\mathrm{k}^2 \cdot \mathrm{E}_{\mathrm{junction}} + 2\mathrm{k}^2(\nu\text{-}1)\mathrm{E}_{\mathrm{cylinder\cdot\,junction}}$$

where ν is the amount of pair layers.

For prime number of layers:

$$E = 2\nu(k^2\text{-}k)E_{cylinder} + (k^2\text{-}k)E_{cylinder} + 2k(2\nu + 1)E_{ellipsis} + 2k^2 \cdot E_{junction}$$

$$+ k^2(4\nu\text{-}2)\frac{E_{cylinder \cdot junction}}{2}$$

$$= 2\nu(k^2\text{-}k)\ E_{cylinder} + (k^2\text{-}k)E_{cylinder} + 2k(2\nu + 1)E_{ellipsis} + 2k^2 \cdot E_{junction}$$

$$+ k^2(2\nu\text{-}1)E_{cylinder \cdot junction}$$

In case of prime number of layers: $\nu = \frac{layers\_number}{2} - 0.5$ (integral number)

For 20 layers, d2=250 μm and d3=150 μm:

$$E_{\ cylinder} = 2\pi 125(c-20) = 250\pi(c-20)$$

$$\mathrm{E}_{junction} = 270^2 - 4 \cdot 25\pi$$

$$\mathrm{E}_{ellipsis} = \pi\frac{270}{2}\cdot\frac{150}{2} = 10125\pi$$

$$E_{cylinder,junction} = 2\pi\frac{150}{2}\left(250 + \frac{8}{100}250\right) = 150\pi \cdot 270 = 40500\pi$$

In summary,

$$E = 500\pi\nu(c-20)(k^2-k) + k\nu\pi 40500 + (270^2 - 4\cdot 25\pi)2k^2 + 2k^2(\nu-1)40500\pi$$

$k = \frac{d+c}{250+c}$ the number of fibers per layer

Similarly, for 27 layers, d2=200 μm and d3=150 μm:

$$E = 400\pi v(k^2 - k)(c-16) + 200\pi(k^2 - k)(c-16) + 16200\pi k(2v+1) + 2k^2(216^2 - 64\pi) + k^2(2v-1)2\pi\frac{150}{2}216 =$$
$$= 400\pi v(k^2 - k)(c-16) + 200\pi(k^2 - k)(c-16) + 16200\pi k(2v+1) + 2k^2(216^2 - 64\pi) + k^2(2v-1)\pi 150 \cdot 216$$

The abovementioned formulas calculate the surface corresponding to a square structure of width and length equal to d (orange square). However, this surface area is much higher than the real one.

Based on the scheme below, the lower and the upper bound of the total area of the cylinder will be the area of the green and red polygon respectively.

Determination of the lower bound of the scaffold area (green polygon).

The angle $T\hat{O}U = \frac{360}{8} = 45^0$

Hence, $\sin(22.5) = \frac{UM}{2} \Rightarrow UM = 2\sin 22.5$

$UT = 4\sin(22.5) = LT$

In the bleu right triangle, we have

$2DT^2 = LT^2$

hence, $2DT^2 = 16\sin^2(22.5) \Rightarrow DT = 2\sqrt{2}\sin(22.5)mm = DL = 1,0823922 \cdot 10^3\,\mu m$

The side of the blue square, then, will be

$TU + 2DT = (4 + 4\sqrt{2})\sin(22.5) = 3,69551813mm = 3,69551813 \cdot 10^3\,\mu m$

Therefore, the lower bound of the area will be given by the formula

$E_{\min} = E_{d=3,69551813 \cdot 10^3\,\mu m} - 2E_{d=DT=1,0823922 \cdot 10^3\,\mu m}$

Determination of the upper bound of the scaffold area (red polygon).

$$\tan(22.5) = \frac{EU}{2} \Rightarrow EU = 2\tan(22.5)$$

Hence, $EG = 4\tan(22.5)$

But AE=AF=GB=BH. Hence,

$$2AE^2 = 16\tan^2(22.5) \Rightarrow AE = 2\sqrt{2}\tan(22.5) = 1.171572875mm = 1.171572875 \cdot 10^3\,\mu m$$

The side of the orange square is $AB = 2AE + EG = (4\sqrt{2}+4)\tan(22.5) = 4mm = 4 \cdot 10^3\,\mu m$

The upper bound of the scaffold area will be equal to the surface of the red polygon,

$$E_{\max} = E_{d=4 \bullet 10^3\,\mu m} - 2E_{d=1,171572875 \bullet 10^3\,\mu m}$$

Finally,

$$E_{d=3,69551813 \bullet 10^3\,\mu m} - 2E_{d=DT=1.0823922 \bullet 10^3\,\mu m} \leq E_{cylinder} \cong E_{d=4 \bullet 10^3\,\mu m} - 2E_{d=1,171572875 \bullet 10^3\,\mu m}$$

The max value is closer to the real one because of the punching process. We consider, therefore, the final surface area of a scaffold to be equal to $\mathrm{E}_{final} \cong E_{d=4 \bullet 10^3\,\mu m} - 2E_{d=1,171572875 \bullet 10^3\,\mu m}$

The aforementioned mathematical theoretical model was validated by comparing its results for 2 scaffolds of specific parameters, whose surface was measured by using μCT. The results are summarized on the table below.

Scaffolds of 27 layers with	Surface by µCT (cm^2)	Surface by model (cm^2)	% Difference
d1=200, d2= 500 and d3=150 µm	4.232	4.129	2.4
d1=200, d2=1100 and d3=150 µm	2.247	2.158	3.9

Figure S1

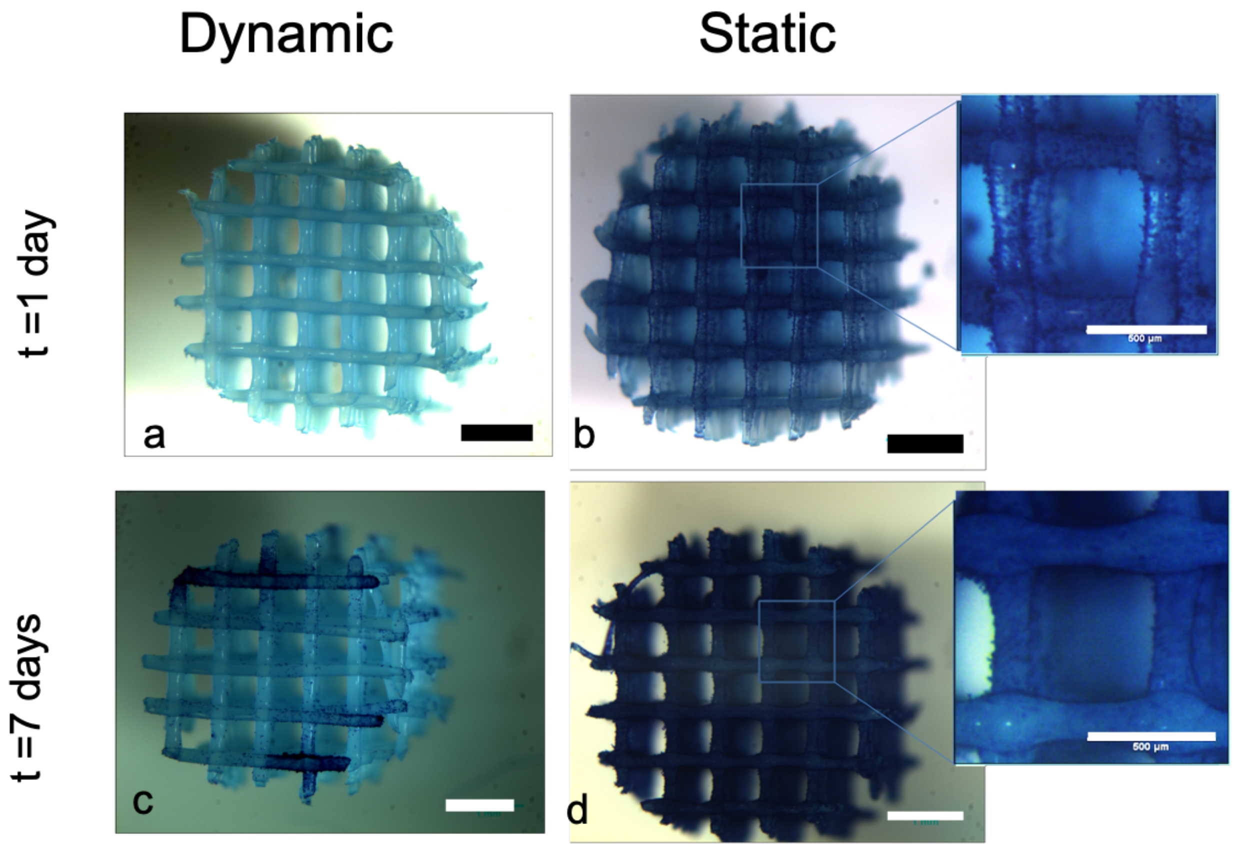

Figure S1. Brightfield images of methylene blue stained scaffolds (with big pores) seeded dynamically (a, c) and statically (b, d). Scaffolds were coated with 100 µg/ml COL-I. Each scaffold was seeded with $0.5 \cdot 10^6$ cells. Representative stereomicroscope images of the scaffolds seeded dynamically at day 1 (a) and at day 7 (c) showing that cells were less confluent in case of dynamic seeding. Representative image of the scaffold seeded statically at day 1 (b) and at day 7 (d) where cells were more efficiently attached. After 7 days of culture, scaffolds were more cell confluent. Scale bar indicates 1 mm (a, b, c, d). A higher magnification illustrating a pore unit is also shown for both time points seeded statically with a scale bar of 500 µm.

Figure S2

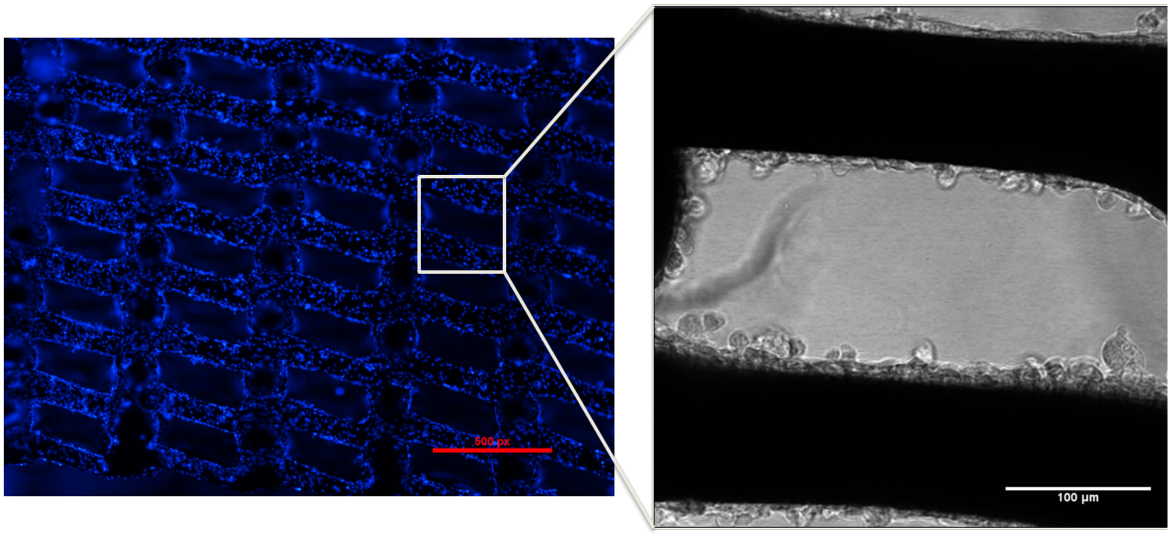

Figure S2. Cell distribution on 3D scaffolds. a) On the left, lateral view of scaffold illustrating homogeneous cell distribution along the scaffold. Cell nuclei are counterstained with DAPI. Scale bar: 1.2 mm. b) On the right, bright field image showing the cell attachment on two parallel fibers. Scale bar: 100 µm. Cells appear to form a monolayer (without any clumps) throughout the whole surface. The stack of parallel fibers (in z direction) is the reason of the dense cell area appearing on the lower fiber. Scaffolds were pre-treated with Sudan Black to hinder their autofluorescence.

Figure S3

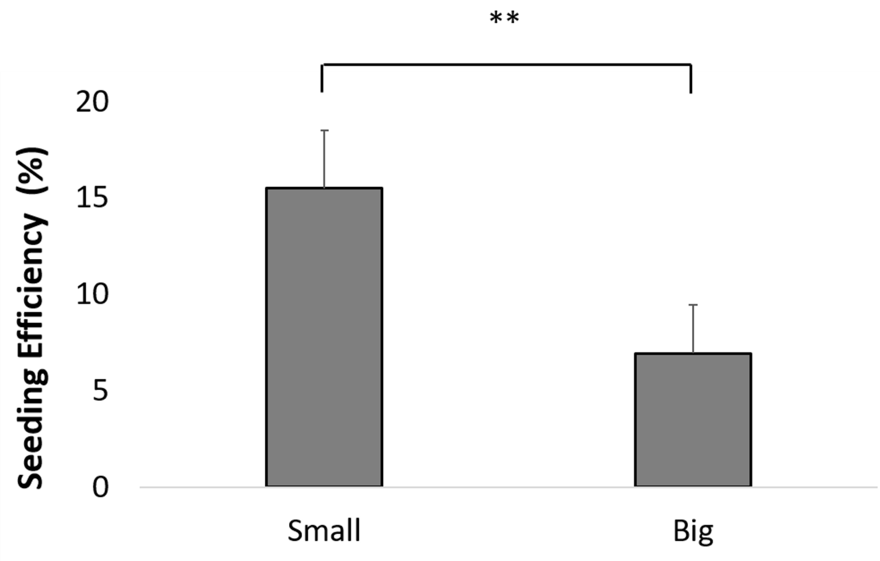

Figure S3. Efficiency of static seeding on scaffolds of different porosities showing a highly significant statistical difference between static and dynamic seeding. Error bars represent mean ± SD, (Student's t-test, P<0.001).
